# Hospitals and Rates

**Published:** 1900-08-18

**Authors:** 


### The Institutional Workshop.

### HOSPITALS AND RATES.

The Select Committee of the House of Commons, which sat in June under the presidency of Mr. T. W. Russell to discuss and hear evidence concerning the exemption of hospitals from rates, may be expected to form a basis for the enunciation of some definite rule on the subject applicable to hospitals and charitable institutions all over the country, which at present are at the mercy of local authorities, some of which are good-natured and some of which are harsh.

The first witness was Mr. A. D. Adrian, legal adviser to the Local Government Board, who put before the Committee a statement of the present position of the law regarding the rating of hospitals. The first record of any legislation on the subject was contained in a fragment of the report of a case which was decided in the first year of Queen Anne's reign. This laid down the rule that hospitals were held rateable like other property. An opposite conclusion was come to in 1760, in the case of St. Luke's Hospital, when the Court of King's Bench, deciding on an appeal from Quarter Sessions, ruled that trustees were not occupiers, and could not be rated as such. Lord Mansfield on this occasion said that no general rule should be deduced from this subject, but that each case should be decided on its own merits; but, as a matter of fact, the ruling in this case became the precedent in subsequent ones, and, in the case of St. Bartholomew's Hospital nine years after, in that of the York Lunatic Asylum in 1832, in that of the Bethlem Hospital in 1847, and in several others, it was used as an argument for the exemption of hospitals from rates. But from 1847 there had been many cases where property held for charitable or beneficial uses had been held chargeable for rates. This still permitted local authorities, if so disposed, to put only nominal assessments on charitable institutions, but the general deduction from such recent cases as that which was decided against the governors of St. Thomas's Hospital in 1875 rather tended to compel authorities to rate such institutions at their real value, and not in trifling sums.

Thus, hospitals are at present of necessity subject to rates, while various other kinds of property are exempt. Among these are included Crown property, parks, post offices, county buildings, churches and chapels, buildings of literary, scientific, and artistic societies, reformatory schools, voluntary schools, volunteer stores, lighthouses, and some barracks.

Mr. C. Burt, chairman of the Central Hospital Committee for London, said that in 1898 a committee of the Council had sent out circulars to 504 hospitals in England and 77 in Scotland, asking their opinion of the rating question. From the English hospitals 356 replies had been received, of which 64 were from London hospitals which share in the grants from the three hospital funds. All the replies showed reasons why hospitals should be relieved from local rates. Mr. Burt stated that the sum paid by London hospitals towards the rates amounted to £21,301 per year, and added that if this payment were remitted the hospitals could take in 4,000 more patients.

The Hon. Sydney Holland, chairman of the London, Poplar, and Tilbury Hospitals, expressed the opinion that the question whether any particular hospital should be exempted should be settled by the Local Government Board. The three hospitals with which he was connected were in a very fortunate position. The London Hospital was exempted in virtue of a local Act, and the Poplar Hospital was rated at only a nominal sum, while both there and at Tilbury so convinced were the guardians that the hospitals really relieved the rates, that they contributed to their funds. Mr. Holland had himself visited 300 patients, and had convinced himself that not more than a quarter of these were in a position to make even the smallest contribution. As he put it, "A man with 30s. a week wages and a cancer in his stomach was at the Poor Law point;" that is, if he could not get the operation necessary to his restoration to health performed in a hospital, he would be compelled to ask for Poor Law aid, as he could not possibly pay the fee for the operation. Mr. Holland argued that the hospitals, as a rule, gave more than an equivalent to the amount demanded from them in the form of rates, and he thought that in these circumstances they ought to be exempted. The point chiefly at issue was, however, whether the hospitals treated, for the most part, patients whose dwelling was within their rating district, or whether they were asking for relief from rates in the interest of strangers, and Mr. Holland declared that at least 5,000 of the 11,000 in-patients treated annually in the London Hospital lived within a mile of its doors.

Sir Edward Hamilton, Assistant Secretary to the Local Government Board, who was examined subsequently, took a different view. He was, on the whole, opposed to exemptions from rates, and pointed out that the tendency of recent Parliamentary action was to reduce as far as possible these exemptions. This had been especially the case since 1870, since which year the only exemption granted had been that to voluntary schools. The justification offered for exempting these schools and not hospitals was that they actually relieved the rates of the locality in which they were situated, whereas many hospitals drew their inmates from a distance. He instanced, as an example of this, the Ventnor Hospital for Consumption. "The ratepayers of Ventnor," he supposed, "directly benefited very little indeed from that hospital. Substantially, the benefit went to people who were not resident in the town, and to omit that property out of the valuation lists would be to inflict a very serious loss upon the ratepayers."

Sir W. Hart Dyke subsequently gave evidence to the same effect. He quoted the case of several institutions, "metropolitan in character and object, though situated near Dartford," such as a home for waifs and strays, convalescent homes, an asylum for idiots, the county asylum, and the City of London Asylum. Sir William admitted that there was a case for the relieving of local hospitals from local rates, but protested that such exemption should not be extended to institutions like the above, where the benefit accrued, not to the district, but to a large town outside the rating area. He brought forward also the case of hospitals for infectious diseases, which were absolutely a detriment to the prosperity of a locality, and protested that it would be a distinct grievance if, in addition to the nuisance which the ratepayers might consider the hospital to be, they were "fined to the amount of the rates upon such an institution." The Marquis of Bristol, who afterwards touched upon the subject of charitable institutions which did not benefit solely the inhabitants of the place in which they were situated, such as the Eastern Counties Asylum for Idiots and Imbeciles, yet claimed that such institutions should be at least free from payment of school and poor rates, from neither of which they benefited.

His Lordship gave some interesting statistics showing the differences of amount asked from kindred institutions in different localities. Thus, the Earlswood Asylum, Surrey, with 700 beds, and about 190 acres of land, was rated at £2,973, and paid £597 annually; whilst the Royal Albert Asylum, with 640 beds and 185 acres of land, was rated at only £1,333, and of that paid only £119 a year. The Eastern Counties Asylum, with 235 beds and 32 acres of land, was rated at £600 and paid £215 annually; while the Western Counties Asylum, with 250 beds and five acres of land, was rated at £561 and paid £79.

Mr. Barton, Commissioner of Valuation in Ireland, brought forward the fact that under the Irish Valuation Act hospitals and charitable institutions in Ireland are exempt from rates. This Act exempts institutions maintained for public, scientific, and charitable purposes exclusively. Yet, though some of the Dublin hospitals have paying beds, and, therefore, might be supposed to be outside the benefits of the Act, they not only escape payment of rates, but receive from the Dublin Corporation £10,000 or £11,000 towards their maintenance. The municipal authorities are, however, now beginning to complain that there are too many exemptions. Mr. Barton, who is now engaged in the revaluation of Belfast, proposes there to exempt totally all hospitals which are open to all without payment of fees, while in institutions in which fees are charged, or where there are paying beds, he would make a separate valuation on a lower basis than that of a shop or a dwelling-house.

The testimony of Mr. Ralph Brocklebank, treasurer of the Liverpool Infirmary, showed that the hospitals in that town had been exempted from rates until last year, when, owing to the inclusion within the city boundaries of Toxteth Park and West Derby, it had been found necessary to assimilate the practice in all three parishes: and thus the Liverpool charitable institutions had lost their privileges. Mr. Brocklebank, like the majority of the witnesses, objected, however, to leaving the question of rating or exemption to the goodwill of local authorities, though these had dealt generously in the case of his own town. These local authorities might at any time change their mind and demand payment of rates, or might decide to withhold any contribution they had given towards the support of a hospital; whereas under a general law the institutions would have absolute security. The witnesses were practically almost unanimous in holding this opinion, whether they recommended total or only partial exemption. The feeling that hospitals should not be asked to pay school rates, from which they benefited nothing, and poor-rates, which their work largely tended to relieve, was general among the witnesses; but the Rev. A. W. Jephson, speaking on behalf of the London School Board, said that the view of that body was that exemptions were unfair to the ratepayers, and he desired that, if possible, Parliament should not only withhold any further exemptions, but should withdraw those it had already granted. The general opinion, however, inclined rather to the view of Mr. Hayward, clerk to the Dartford Board of Guardians, who was not opposed to the principle of exemption, but demanded that a condition of it should be that it could be shown that the benefit to the neighbourhood in which the hospital was situated was of greater pecuniary value than the rates that might be demanded.

